# Astaxanthin protects against environmentally persistent free radical-induced oxidative stress in well-differentiated respiratory epithelium

**DOI:** 10.1016/j.redox.2025.103542

**Published:** 2025-02-09

**Authors:** Ayaho Yamamoto, Peter D. Sly, Lavrent Khachatryan, Nelufa Begum, Abrey J. Yeo, Paul D. Robinson, Stephania A. Cormier, Emmanuelle Fantino

**Affiliations:** a Child Health Research Centre, The University of Queensland, South Brisbane, Queensland, 4101, Australia; b Department of Chemistry, Louisiana State University, Baton Rouge, LA, 70803, United States; c Centre for Clinical Research, The University of Queensland, Herston, Queensland, 4006, Australia; d Department of Biological Sciences, and Pennington Biomedical Research Center, Louisiana State University, Baton Rouge, LA, 70803, United States

**Keywords:** Air-liquid interface culture, Air pollution, Antioxidant, Mitochondria, Mitochondrial reactive oxygen species, Mucus

## Abstract

Environmentally persistent free radicals (EPFRs) are combustion products present in substantial numbers on atmospheric particulate matter with half-lives of days to years. The mechanisms linking EPFR exposure and respiratory diseases are unclear, but likely involve oxidative stress. We investigated the mechanisms by which EPFR exposure impact on well-differentiated primary human nasal epithelial cells from subjects sensitive or resistant to oxidant stressors, cultured at an air-liquid interface. We found that EPFR exposure induced mitochondrial reactive oxygen species (mtROS) production; increased mitochondrial DNA copy number; down-regulated mucus production gene, Mucin-5AC (*MUC5AC*); up-regulated detoxifying gene, cytochrome P450 1A1 (*CYP1A1*), nuclear factor erythroid 2-related factor 2 (NRF2)-regulated antioxidant pathways including Sirtuin 1 (*SIRT1*)-Forkhead box O3 (*FOXO3*), mitophagy, PTEN-induced kinase 1 (*PINK1*), apoptosis, cyclin-dependent kinase inhibitor p21 (*p21*), and inflammation, C–C motif chemokine ligand 5 (*CCL5*). These results indicate that the well-differentiated respiratory epithelium can respond and activate redox reactions when exposed to sublethal concentrations of EPFRs. Increased susceptibility to EPFR exposure is conferred by failure to upregulate the mucin gene, *MUC5AC*, expression. Pre-treatment with astaxanthin prevented most of the negative impacts caused by EPFRs. Our results demonstrate that EPFRs can induce oxidative stress and cause damage to respiratory epithelium. A dietary antioxidant, astaxanthin, protected cells from EPFR-induced oxidant stress.

## Highlights

• EPFRs damaged the respiratory epithelium by inhibiting mucus production.
• EPFR-induced oxidative stress activated defense mechanisms to limit cellular damage.
• Susceptibility to EPFR exposure varied between healthy human adults.
• Astaxanthin, an antioxidant, prevented most of the negative impacts caused by EPFRs.
• NRF2-mediated antioxidant pathways play an important role in response to EPFRs.

## Graphical abstract

**Figure:**
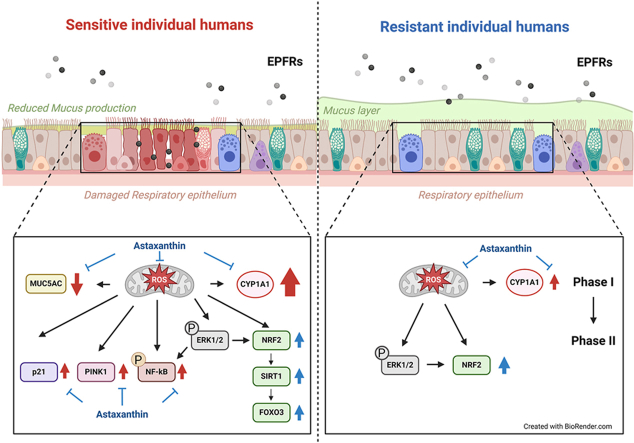

**Table:** List of abbreviations

AHR	Aryl hydrocarbon receptor
ALI	Air-liquid interface
AOX1	Aldehyde oxidase 1
APOE	Apolipoprotein E
CCL5	C–C motif chemokine ligand 5
CYP1A1	Cytochrome P450 1A1
CuO	Copper nitrate hemipentahydrate
DCB230	1,2-dichlorobenzene
EPFRs	Environmentally persistent free radicals
ERK	Extracellular signal-regulated kinase
FOXO3	Forkhead box O3
GAPDH	Glyceraldehyde 3-phosphate dehydrogenase
GPX2	Glutathione peroxidase 2
GPX3	Glutathione peroxidase 3
GSH	Glutathione
HMOX1	Heme oxygenase 1
H_2_O_2_	Hydrogen peroxide
MAPK	Mitogen-activated protein kinases
MitoQ	Mitoquinone
mtDNAcn	Mitochondrial DNA copy number
mtROS	Mitochondrial reactive oxygen species
MUC5AC	Mucin-5AC
ND1	NADH-ubiquinone oxidoreductase chain 1
ND5	NADH-ubiquinone oxidoreductase chain 5
NECs	Nasal epithelial cells
NF-κB	Nuclear factor kappa-light-chain-enhancer of activated B cells
NOS2	Nitric oxide synthase 2
NRF2	Nuclear factor erythroid 2-related factor 2
NQO1	NAD(P)H dehydrogenase (quinone 1)
OS	Oxidative stress
P21	Cyclin-dependent kinase inhibitor p21 (CDKN1A)
PINK1	PTEN-induced kinase 1
PM	Particulate matter
SEPP1	Selenoprotein P
SERPINA1	The gene that codes for Alpha-1 antitrypsin production
SIRT1	Sirtuin 1
SLCO2B1	Solute carrier organic anion transporter family member 2B1
SOD2	Superoxide dismutase 2
SPINK1	Serine protease inhibitor Kazal-type 1
SQSTM1	Sequestosome 1
SRXN1	Sulfiredoxin 1
TEER	Transepithelial electrical resistance
TRAP	Traffic related air pollution

## 1 Introduction

Air pollution causes around seven million premature deaths each year worldwide [1]. Both indoor and outdoor air pollution negatively affect respiratory health [2]. Airborne particulate matter (PM) is associated with many acute and chronic respiratory diseases, increases severity of respiratory viral infections, increased mortality, causes a public health issue [3,4]. Traffic related air pollution (TRAP) exposure during childhood is associated with an increased risk of severe respiratory infections [5,6], lower lung function and reduced lung growth [7,8], wheezing illnesses, incident asthma, and asthma exacerbations [9,10]. Acute TRAP exposure can decrease glutathione (GSH) in healthy adults [11]. Children exposed to high levels of TRAP in Mexico City had increased levels of malondialdehyde, an oxidative stress (OS)-induced product of lipid peroxidation, in exhaled breath condensates [12]. Subjects with “null” or reduced function mutations in antioxidant defence genes, showed increased susceptibility to TRAP exposure [13], and asthma was more likely in children with TRAP exposure if they had increased expression of the redox-sensitive transcription factor, nuclear factor erythroid 2-related factor 2 (NRF2) gene [14]. Evidence is accumulating that TRAP exposure induces OS in humans, however, studies aimed at increasing antioxidant defence to prevent adverse effects of TRAP exposure are lacking [15,16].

Environmental exposures and chronic lung diseases increase cellular reactive oxygen species (ROS) levels which is known as oxidative stress [17], this may result in damage to cell structures. ROS are chemically reactive chemical species containing oxygen such as hydrogen peroxide (H_2_O_2_), superoxide anion (O_2_^●−^) and hydroxyl radicals (•OH). In living organisms, ROS are produced as a natural by-product of cellular metabolism, which is associated with homeostasis and cell signalling [18].

Combustion related environmental pollutants such as diesel exhaust, burning fossil-fuel, cigarette smoke, and wildfire smoke contain the combustion by-product, environmentally persistent free radicals (EPFRs) [19,20]. EPFRs are present in substantial numbers on atmospheric PM, with half-lives of days to years [21]. A previous study showed that the redox cycle of PM containing EPFRs can produce highly reactive hydroxyl radicals (•OH) and that this activity can persist in the lungs of living organisms [22]. EPFRs generated from diesel exhaust can last in the environment for several years and interact with living organisms [23]. Epidemiological evidence shows EPFR exposure causes negative impacts in humans [24], however, the precise mechanisms linking EPFR exposure to adverse respiratory outcomes remains unclear. As the nose is the point of first contact with the ambient environment, it is therefore important to understand the impact of air pollution, especially EPFRs on nasal epithelium. The aim of this study was to understand the mechanisms by which EPFR exposure induce adverse impacts on respiratory epithelium. We have previously shown that individuals can be classified as “sensitive” or “resistant” to oxidant stimuli, based on the concentration of H_2_O_2_ required to induce epithelial leak [18]. The present study includes both sensitive and resistant individuals.

## 2 Materials and methods

### 2.1 Cell collection

Healthy non-atopic, non-smoking adult volunteers between 20 and 45 years of age were recruited, and primary human nasal epithelial cells (NECs) collected using Rhino-Pro Nasal Curette (Arlington Scientific, UT, USA). Ethics approval: No.#UQ2017000520; HREC61894; UQ2020001742. The collected cells were grown as submerged cultures in PneumaCult™-Ex Medium (STEMCELL Technologies, BC, Canada) until they reached passage 2.

### 2.2 Air-liquid interface culture

Cells were seeded (4∗10^4^ cells/insert) onto 6.5 mm Transwell® with 0.4 μm Pore Polyester Membrane Inserts (Corning, NY, USA) and differentiated with PneumaCult-ALI Medium (STEMCELL Technologies). ALI conditions were maintained for at least three weeks until cells were fully differentiated.

### 2.3 Subject screening for sensitive or resistant to oxidative stress

As in our previous study [18], we have classified subjects as being sensitive or resistant to OS based on the susceptibility to H_2_O_2_ exposure on epithelial response. The same grouping was used in this study to examine the differential mechanisms.

### 2.4 Environmentally persistent free radicals (EPFRs)

EPFRs-1,2-dichlorobenzene (DCB230), attached to ultrafine (<0.2 μm) PM, were used in this study. Particle-associated persistent free radical (PFR) is formed through physisorption, chemisorption of HX, radical formation by electron transfer and a reduced metal. More details of PFR synthesis have been described in previous studies [25,26]. In short, silica gel was soaked with 5 % copper nitrate hemipentahydrate (CuO). The 5 % CuO supported on silica was dosed with 1,2-dichlorobenzene in a custom made vacuum exposure system at 230 °C [26]. Any physisorbed dosant was removed and the particles cooled to room temperature. Electron paramagnetic resonance was used to confirm the presence of free radicals. EPFRs-DCB230 were then aliquoted into vacuum-sealed glass tubes, 10mg/vial (3.24 × 10^17^ spins/g).

10 mg of EPFRs-DCB230 was suspended in 1 ml of HBSS containing 0.02 % Tween80, the suspended solution was then sonicated for 4 min on ice, using cycles of 30 s on and 30 s off and adjusted the amplitude of the probe sonicator to 50 %. The suspension was then vortexed for 1 min before proceeding with exposure.

EPFRs have been shown to cause cellular damage within 3–6 h [27], thus, the epithelium in ALI culture were exposed to 1 mg/cm^2^ (1.1 × 10^16^ spins/g) or 2 mg/cm^2^ EPFRs-DCB230 or silica vehicle control for 4 h.

### 2.5 Antioxidants pretreatment

As previously reported [28], pretreatment with resveratrol and astaxanthin prevented H_2_O_2_-induced OS, thus, they were used in the present study. Mitoquinone (MitoQ) was also used as a control antioxidant. When the NECs were fully differentiated, selected wells were treated with 20 μM resveratrol (R5010; Sigma-Aldrich, MI, USA), or 20 μM astaxanthin (SML0982; Sigma-Aldrich, MI, USA) or 1 μM MitoQ (29317; Cayman Chemical, MI, USA) added to the basal media for 24 h prior to EPFR exposure.

### 2.6 Reagents and assay kits

Fluorescein isothiocyanate-dextran (Sigma-Aldrich, MI, USA); CytoTox 96® Non-Radioactive Cytotoxicity Assay (Promega Corporation, WI, USA); MitoSOX™ Red mitochondrial superoxide indicator (Invitrogen™, CA, USA); Hoechst 33342 (Invitrogen™, CA, USA); PureLink™ Genomic DNA Mini Kit (Invitrogen™, CA, USA); Human Mitochondrial DNA Monitoring Primer Set (Takara Bio, CA, USA); TB Green® Premix Ex Taq™ II (Tli RNase H Plus) (Takara Bio, CA, USA); TRIzol™ reagent (Invitrogen™, CA, USA); RNeasy Mini Kit (Qiagen, Hilden, Germany); High Capacity cDNA Reverse Transcription Kit (Applied Biosystems™, CA, USA); RT^2^ First Strand Kit, RT^2^ SYBR Green ROX qPCR Mastermix, and RT^2^ Profiler PCR Array Human Oxidative Stress Plus (Qiagen, Hilden, Germany); NRF2 Antibody (Santa Cruz Biotechnology, TX, USA).

### 2.7 Quantitative reverse transcription PCR (qRT-PCR)

qPCR was performed with Taqman primers (Applied Biosystems™, CA, USA): *CYP1A1* (Hs00153120_m1); *MUC5AC* (Hs00873651_mH); *SIRT1* (Hs01009005_m1); *CDKN1A* (*p21*; Hs99999142_m1); *FOXO3* (Hs00818121_m1); *PINK1* (Hs00260868_m1) with FAM dye, and housekeeping gene Eukaryotic 18S rRNA Endogenous Control (4319413E) with VIC dye.

Quantification of gene expression was performed using a ViiA™ 7 Real-Time PCR System (Applied Biosystems, MA, USA) and the relative mRNA expression levels were normalized to 18s using the 2^−ΔΔCq^ methods.

The data from PCR array were analyzed by QIAGEN Ingenuity Pathway Analysis software [29], and gene-gene interaction was determined using GeneMANIA database [30].

### 2.8 Western blot

Equal quantities of protein were loaded to Bolt™ 4–12 %, Bis-Tris Gel (Thermo Fisher Scientific, MA, USA), electrophoresed at 200V for 30 min, transferred to Immobilon-P PVDF membrane (Merck KGaA, Germany). Membranes were blocked with 5 % BSA blocking buffers for 1 h at room temperature and incubated with primary antibodies, phospho-p44/42 MAPK (Erk1/2) (1:1000), p44/42 MAPK (Erk1/2) (1:1000), phospho–NF–kappaB p65 (Ser536) (1:1000), NF-κB p65 (D14E12) (1:1000), GAPDH (1:1000), NQO1 (1:1000) (Cell signaling, MA, USA); or GPX2 (1:1000) (Invitrogen™, CA, USA) in blocking buffer at 4 °C overnight. The following day, membranes were incubated with appropriate HRP-linked secondary antibodies at room temperature for 1 h, detected using the chemiPRO chemiluminescence imaging system (Cleaver Scientific, Warwickshire, UK) and quantiﬁed by ImageJ (National Institutes of Health, Bethesda, MD).

### 2.9 Statistical analysis

All the graphs were plotted using GraphPad Prism 9.5.1 (GraphPad Inc., CA, USA) and were expressed as the mean ± standard deviation.

Kruskal-Wallis rank test with Dunn's multiple-comparison test was performed to compare different conditions. Two-sample Wilcoxon rank-sum (Mann-Whitney) test was used to compare sensitive and resistant groups. *P* < 0.05 was considered to indicate a statistically significant difference.

## 3 Results

### 3.1 EPFR exposure caused a decrease in cell integrity with partial reversal by anti-oxidants

**Fig. 1:**
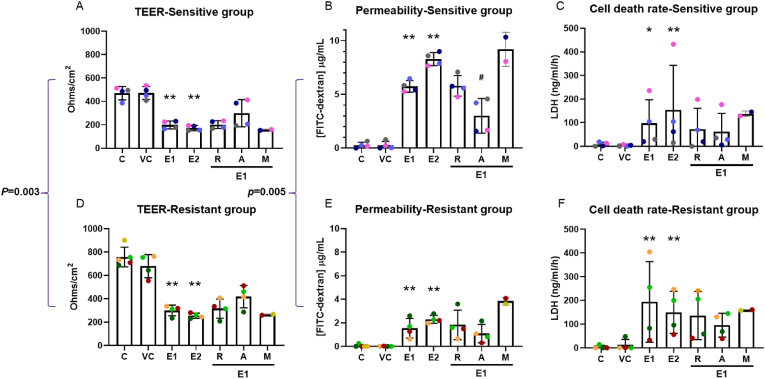
EPFR exposure decreased cell integrity in both sensitive (A, B, C) and resistant (D, E, F) groups. Data are shown after 4-h EPFR exposures. 1 mg/cm^2^ and 2 mg/cm^2^ EPFR exposure caused a decrease in TEER (A, D), an increase in permeability (B, E), and in cell death (C, F). Astaxanthin prevented these effects. Significant differences were observed between the sensitive and resistant groups in response to EPFRs. Data presented as mean ± SD (n = 4 in each group; ∗*p* < 0.05; ∗∗*p* < 0.01, compared with silica vehicle control; ^#^*p* < 0.05, compared with 1 mg/cm^2^ EPFRs). C: control; VC: vehicle control; E1: 1 mg/cm^2^ EPFRs; E2: 2 mg/cm^2^ EPFRs; R: resveratrol; A: astaxanthin; M: MitoQ. A single colour was used in all figures to represent each separate donor.

Cell integrity was measured following EPFR exposure (Fig. 1). There was a dose-dependent decrease in transepithelial electrical resistance (TEER) (*p* = 0.018, *p* = 0.015, respectively; Fig. 1A and D) and a dose-dependent increase in permeability (*p* = 0.007, *p* = 0.015, respectively; Fig. 1B and E) and cell death (*p* = 0.024, *p* = 0.039, respectively; Fig. 1C and F) in both sensitive and resistant groups. There was a significant difference between the sensitive and resistant groups in EPFR-induced reduction in TEER (*p* = 0.003; Fig. 1A and D), increase in permeability (*p* = 0.005; Fig. 1B and E), but not in cell death (*p* = 0.224; Fig. 1C and F).

Astaxanthin, and to a lesser extent resveratrol, pre-treatment reduced the effects of EPFRs on TEER (overall *p* = 0.08), permeability (overall *p* = 0.17), and cell death (overall *p* = 0.23), although these effects did not reach statistical significance and did not completely prevent the effects (Fig. 1).

### 3.2 EPFR exposure induced mtROS production, prevented by resveratrol, astaxanthin or MitoQ

**Fig. 2:**
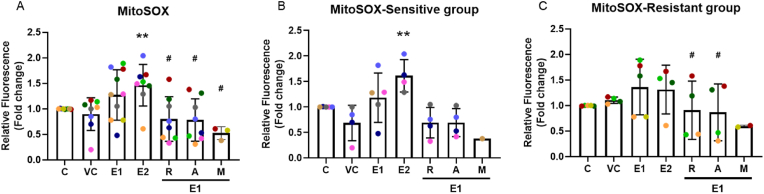
EPFR exposure induced mtROS that was prevented by resveratrol, astaxanthin or MitoQ. After 4 h EPFR exposure, MitoSOX staining was performed, confocal images were captured and quantified. EPFR exposure caused an increase in mtROS, while the effect was inhibited by 24-h pre-treatment of 20 μM resveratrol, 20 μM astaxanthin or 1 μM MitoQ. There was no difference between the sensitive and resistant groups. Data presented as the mean ± SD (n = 4 in each group; ∗*p* < 0.05; ∗∗*p* < 0.01, compared with silica vehicle control; ^#^*p* < 0.05, compared with 1 mg/cm^2^ EPFRs). C: control; VC: vehicle control; E1: 1 mg/cm^2^ EPFRs; E2: 2 mg/cm^2^ EPFRs; R: resveratrol; A: astaxanthin; M: MitoQ. A single colour was used in all figures to represent each separate donor.

To measure the levels of mitochondrial superoxide generated, a MitoSOX assay was performed to determine whether EPFRs induced mtROS production. Results in Fig. S2 showed images from a representative sensitive subject (A) and a resistant subject (B). EPFR exposure increased MitoSOX labelling, and this was prevented by pre-treatment with resveratrol, astaxanthin, or MitoQ (Fig. 2A). There was a dose-dependent rise in mtROS generation following 1 mg/cm^2^ and 2 mg/cm^2^ EPFR exposure in the sensitive group (*p* = 0.046, *p* = 0.003, respectively; Fig. 2B), but not in the resistant group (*p* = 0.120, *p* = 0.242, respectively; Fig. 2C), with no differences between the groups (*p* = 0.248). 20 μM resveratrol and 20 μM astaxanthin pre-treatment prevented the increase in mtROS generation in the resistant (*p* = 0.049, *p* = 0.037, respectively; Fig. 2C) group and a trend of inhibition in the sensitive (*p* = 0.063, *p* = 0.060, respectively; Fig. 2B) group.

### 3.3 EPFR exposure increased mitochondrial DNA copy number

**Fig. 3:**
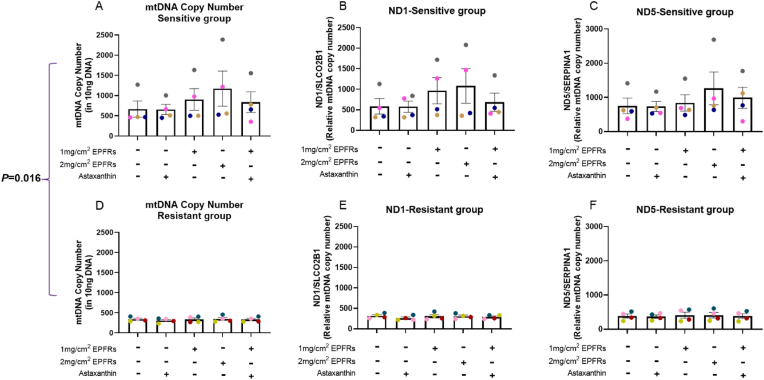
EPFR exposure caused an increase in mtDNA copy number. The relative mtDNA copy number was quantified using nuclear DNA content as a standard in the sensitive (A) and the resistant (D) groups. The data (A, D) are shown as the mean of ND1/SLCO2B1 (B, E) and ND5/SERPINA1 (C, F) in each group. Data presented as the mean ± SEM (n = 4 in each group). A single colour was used in all figures to represent each separate donor.

Mitochondria play an important role in response to oxidant exposure and are involved in cell homeostasis via multiple mechanisms. EPFR exposure caused a dose-dependent increase in mitochondrial DNA copy number (mtDNAcn) following 1 mg/cm^2^ and 2 mg/cm^2^ EPFR exposure in the sensitive group (*p* = 0.074, *p* = 0.074, respectively; Fig. 3A, B and C), but not in the resistant group (*p* = 0.387, *p* = 0.875, respectively; Fig. 3D, E and F). Although these effects did not reach statistical significance in the group data, each individual subject showed similar trend. A significant difference was observed in baseline (control) mtDNAcn between the sensitive and resistant groups (*p* = 0.016; Fig. 3A and D).

### 3.4 EPFR exposure increased *CYP1A1* and reduced *MUC5AC* mRNA expression in the sensitive group and was prevented by astaxanthin

**Fig. 4:**
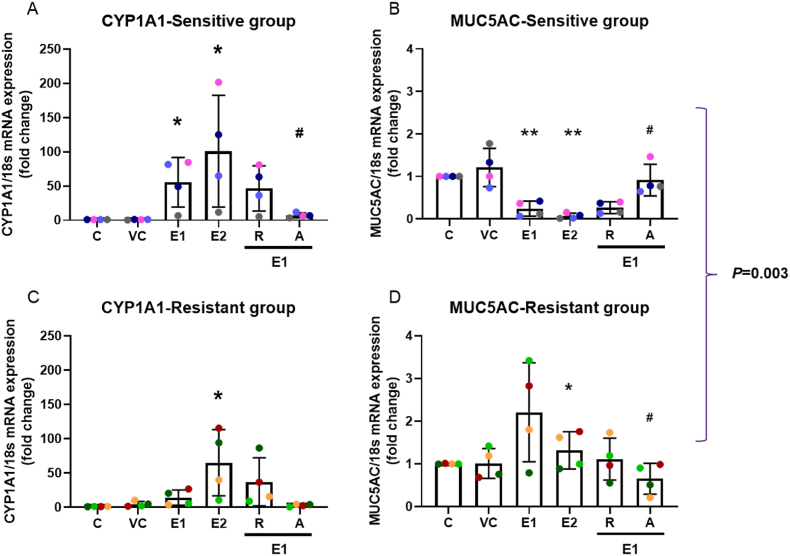
EPFR exposure upregulated *CYP1A1* and downregulated *MUC5AC* mRNA expression and was prevented by astaxanthin. EPFR exposure significantly increased *CYP1A1* mRNA expression in a dose-dependent manner in both sensitive (A) and resistant (C) groups. *MUC5AC* mRNA expression decreased following EPFR exposure in the sensitive group (B), but slightly increased in the resistant group (D). Astaxanthin prevented these effects. A significant difference was observed between the sensitive and resistant groups. Data presented as the mean ± SD (n = 4 in each group; ∗*p* < 0.05; ∗∗*p* < 0.01, compared with silica vehicle control; ^**#**^*p* < 0.05, compared with 1 mg/cm^2^ EPFRs). C: control; VC: vehicle control; E1: 1 mg/cm^2^ EPFRs; E2: 2 mg/cm^2^ EPFRs; R: resveratrol; A: astaxanthin. A single colour was used in all figures to represent each separate donor.

EPFR exposure upregulated *CYP1A1* mRNA expression in both sensitive (*p* = 0.029 (E1), *p* = 0.046 (E2); Fig. 4A) and resistant (*p* = 0.053 (E1), *p* = 0.039 (E2); Fig. 4B) groups in a dose-dependent manner. Astaxanthin prevented the increase (*p* = 0.032, *p* = 0.069, respectively; Fig. 4A and C) but resveratrol did not (*p* = 0.262, *p* = 0.125, respectively; Fig. 4A and C).

The epithelial mucus layer is the first line defence against environmental exposure; maintaining the mucus layer is critical for protecting the airway epithelium, thus, *MUC5AC* mRNA expression was examined following EPFR exposure. The pattern of change in the mucin gene *MUC5AC* expression following 1 mg/cm^2^ or 2 mg/cm^2^ EPFR exposure differed between the sensitive and resistant groups. EPFR exposure reduced expression in the sensitive group (*p* = 0.007, *p* = 0.001, respectively; Fig. 4B), while *MUC5AC* mRNA expression was slightly increased with EPFR exposure in the resistant group (*p* = 0.058, *p* = 0.048, respectively; Fig. 4D). A significant difference was observed between the sensitive and resistant groups (*p* = 0.003), however, there was no difference in baseline *MUC5AC* mRNA expression between the groups.

Astaxanthin pre-treatment prevented the decline in *MUC5AC* expression in the sensitive group following 1 mg/cm^2^ EPFR exposure (*p* = 0.03; Fig. 4B), whereas resveratrol had no effect (*p* = 0.38; Fig. 4B). Astaxanthin (*p* = 0.03), but not resveratrol (*p* = 0.10), also prevented the increase in *MUC5AC* mRNA expression following EPFR exposure in the resistant group (Fig. 4D).

### 3.5 EPFR exposure increased mRNA expression of *SIRT1*, *FOXO3*, *PINK1*, and *p21*, and was inhibited by astaxanthin in the sensitive individuals

**Fig. 5:**
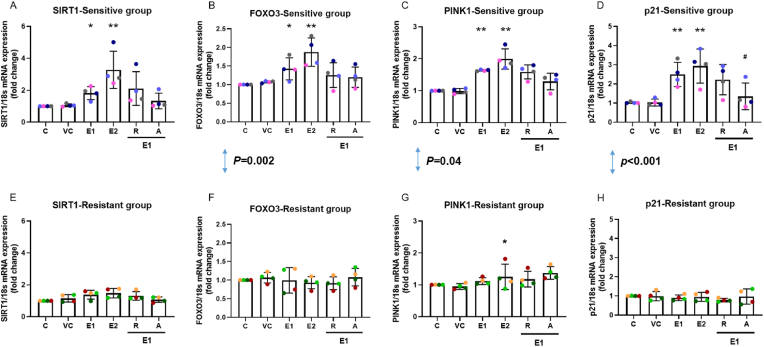
EPFR exposure impacted on antioxidant, mitophagy, and apoptosis pathways. EPFR exposure caused a rise in *SIRT1* (A), *FOXO3* (B), *PINK1* (C), and *p21* (D) mRNA expression in the sensitive group, while EPFR exposure only slightly increased in *PINK1* mRNA expression in the resistant group (G). There was a significant difference observed for *FOXO3* (B, F), *PINK1* (C, G), and *p21* (D, H), between the sensitive and resistant groups. Pre-treatment with astaxanthin significantly inhibited *p2*1 mRNA expression (D) with a trend for reduction in *SIRT1* (A), *FOXO3* (B) and *PINK1* (C) in the sensitive group, while no change in mRNA expression with resveratrol pre-treatment (A, B, C, D). Data presented as the mean ± SD (n = 4 in each group; ∗*p* < 0.05, ∗∗*p* < 0.01, compared with silica vehicle control; ^**#**^*p* < 0.05, compared with 1 mg/cm^2^ EPFRs). C: control; VC: vehicle control; E1: 1 mg/cm^2^ EPFRs; E2: 2 mg/cm^2^ EPFRs; R: resveratrol; A: astaxanthin. A single colour was used in all figures to represent each separate donor.

To investigate the SIRT1 signalling pathway, mRNA expression of several key players including *SIRT1*, *FOXO3*, *PINK1*, and *p21* were assessed following EPFR exposure (Fig. 5). Within the sensitive group (Fig. 5A, B, C and D), EPFR exposure caused dose-dependent increases in mRNA expression of *SIRT1* (*p* = 0.058 (E1), *p* = 0.001 (E2); Fig. 5A), *FOXO3* (*p* = 0.050 (E1), *p* = 0.003 (E2); Fig. 5B), *PINK1* (*p* = 0.001 (E1), *p* = 0.001 (E2); Fig. 5C), and *p21* (*p* = 0.004 (E1), *p* = 0.005 (E2); Fig. 5D). Within the resistant group, an increase in *PINK1* mRNA expression was also observed (*p* = 0.058 (E1), *p* = 0.048 (E2); Fig. 5G), following 1 mg/cm^2^ and 2 mg/cm^2^ EPFR exposure however no significant changes in expression of *SIRT1* (*p* = 0.085, *p* = 0.058, respectively; Fig. 5E), *FOXO3* (*p* = 0.442, *p* = 0.246, respectively; Fig. 5F), or *p21* (*p* = 0.422, *p* = 0.347, respectively; Fig. 5H) were observed. When comparing between the sensitive and resistant groups, EPFR exposure resulted in significant difference in the expression of *FOXO3* (*p* = 0.002), *PINK1* (*p* = 0.04), and *p21* (*p* < 0.001), but not in *SIRT1* (*p* = 0.07).

20 μM astaxanthin pre-treatment significantly inhibited the EPFR-induced upregulation of *p21* expression in the sensitive group (*p* = 0.04; Fig. 5D). There was also a downward trend observed in the expression of *SIRT1* (*p* = 0.17; Fig. 5A), *FOXO3* (*p* = 0.18; Fig. 5B) and *PINK1* (*p* = 0.08; Fig. 5C) with astaxanthin treatment in the sensitive group. However, 20 μM resveratrol pre-treatment did not show a change in those mRNA expressions (*p* = 0.36, *p* = 0.40, *p* = 0.26, *p* = 0.46, respectively). Neither astaxanthin nor resveratrol had any effects on mRNA expression in the resistant group.

### 3.6 Similarities and differences in effects of EPFR and H_2_O_2_ exposure on expression of genes involved in oxidative stress

The responses to EPFR exposure in the sensitive and resistant individuals were quite different. Individuals were classified as sensitive or resistant by the impact of H_2_O_2_ on cell integrity. While EPFR exposure also induces OS, the mechanisms may differ between these two oxidant stressors. To determine which signalling pathways may be involved in the response to EPFR, RT^2^ Profiler PCR Array Oxidative Stress Gene Expression was performed. The PCR array contains 84 OS related genes. Two subjects from each group were assessed following 1 mg/cm^2^ EPFR exposure or 50 mM H_2_O_2_ (oxidant control). Fig. S3 shows a list of up and down-regulated genes in the sensitive (A) and resistant (B) groups.

In the sensitive group, 8 genes were up-regulated with both EPFR and H_2_O_2_ exposure, 9 genes were exclusively up-regulated following EPFR exposure, and 4 genes were exclusively up-regulated with H_2_O_2_ exposure (Fig. S3A). 9 genes were down-regulated with both EPFR and H_2_O_2_ exposure, 6 genes were exclusively down-regulated after EPFR exposure, and 4 genes were exclusively down-regulated with H_2_O_2_ exposure (Fig. S3A).

In the resistant group, 6 genes were up-regulated with both EPFR and H_2_O_2_ exposure, 11 genes were exclusively up-regulated following EPFR exposure, and 1 gene was exclusively up-regulated with H_2_O_2_ exposure (Fig. S3B). 1 gene was exclusively down-regulated following EPFR exposure, and 9 genes were exclusively down-regulated with H_2_O_2_ exposure (Fig. S3B).

*AOX1*, *SRXN1*, *HMOX1*, *SPINK1* were up-regulated with EPFR and H_2_O_2_ exposure in both sensitive and resistant groups. Greater fold changes were observed in the sensitive compared with the resistant group and more genes were involved with response to the exposures in the sensitive group. *SPINK1* was remarkably up-regulated with EPFR and H_2_O_2_ exposure in the sensitive (40-fold, 24.7-fold, respectively) and resistant (14.2-fold, 4.1-fold, respectively) groups. EPFR exposure induced significant up-regulation in *HMOX1* by 5.3-fold in the sensitive and 4.8-fold in the resistant group.

*APOE*, *GPX3*, *SOD2* and *SQSTM1* were commonly up-regulated following EPFR exposure in both groups. EPFR exposure induced *APOE* and *SOD2* up-regulation in the sensitive (2.4-fold, 2.2-fold, respectively), and resistant (2.4-fold, 2.3-fold, respectively) groups.

*SEPP1* was down-regulated with both EPFR and H_2_O_2_ exposure in the sensitive group (−7.35-fold, −5.26-fold, respectively). In the resistant group, *SEPP1* was up-regulated with EPFR exposure (2.1-fold) and down-regulated with H_2_O_2_ exposure (−2.1-fold). *CCL5* was up-regulated with EPFR exposure (8.4-fold) and down-regulated with H_2_O_2_ exposure (−2.3-fold) in the sensitive group. *NOS2* was up-regulated with EPFR exposure (2.3-fold) and significantly down-regulated with H_2_O_2_ exposure (−22.5-fold) in the resistant group.

The genes involved in response to EPFRs were analyzed with its function and gene-gene interaction (Fig. S4), and clustered (Fig. S5).

### 3.7 EPFR exposure increased ERK and NF-κB phosphorylation and suppressed NQO1 protein expression

**Fig. 6:**
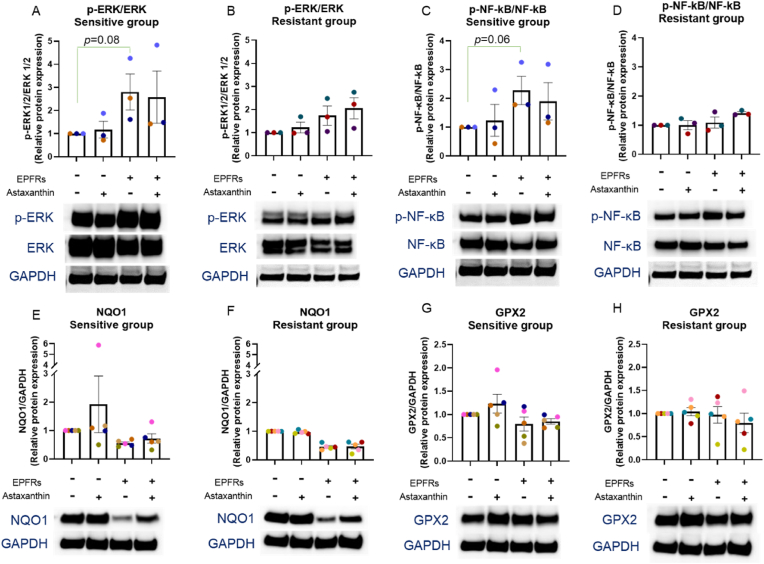
EPFR exposure increased ERK and NF-κB phosphorylation and suppressed NQO1 e**xpression.** EPFR exposure caused a rise in ERK phosphorylation in both sensitive (A) and resistant (B) groups, NF-κB phosphorylation in the sensitive group (C), but no change in NF-κB phosphorylation in the resistant group (D). There was a reduction in NQO1 following EPFR exposure (E and F), but no change in GPX2 (G and H). Data presented as the mean ± SEM (n = 3–5 in each group). A single colour was used in all figures to represent each separate donor.

To further understand the mechanisms, the protein that related to MUC5AC expression was measured. EPFR exposure increased ERK and NF-κB phosphorylation in the sensitive group (*p* = 0.08, *p* = 0.06, respectively; Fig. 6A and C), however, did not reach statistical significance. In the resistant group, there was a trend of increasing ERK phosphorylation with EPFR exposure (Fig. 6B), but no effect on NF-κB phosphorylation (Fig. 6D). EPFR exposure decreased NQO1 expression in both sensitive (*p* = 0.06; Fig. 6E) and resistant (*p* = 0.06; Fig. 6F) groups, while no effect on GPX2 expression in both sensitive and resistant group (Fig. 6G and H).

### 3.8 EPFR exposure increased NRF2 nuclear translocation

**Fig. 7:**
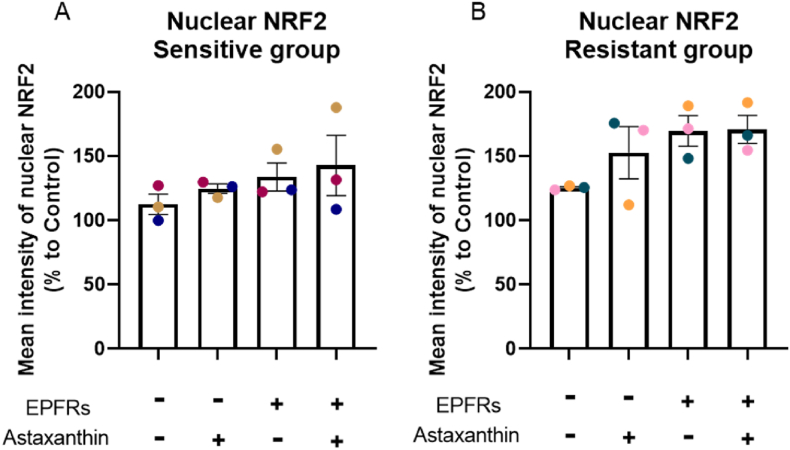
EPFR exposure increased NRF2 nuclear translocation. EPFR exposure caused an increase trend of NRF2 nuclear translocation in both sensitive (A) and resistant (B) groups with grater magnitude in the resistant group. Data presented as the mean ± SEM (n = 3 in each group). A single colour was used in all figures to represent each separate donor.

Following the ingenuity pathway analysis (IPA, QIAGEN), NRF2-mediated oxidative stress response was the top canonical pathways in both sensitive (*p* = 2.06 × 10^−11^) and resistant (*p* = 2.74 × 10^−10^) groups, with higher activation z-score in the resistant group (2.236) compared to the sensitive group (1.633). EPFR exposure increased NRF2 nuclear translocation in the resistant group (*p* = 0.06; Fig. 7B), and a tendency of rise in the sensitive group (Fig. 7A). There was a trend of increasing NRF2 translocation with astaxanthin pretreatment in both groups. Astaxanthin pretreatment had no effect on EPFR increased NRF2 translocation, but there was a significant increase when compared to control (*p* = 0.05). Results in Fig. S6 showed images from a representative sensitive subject (A) and a resistant subject (B).

## 4 Discussion

According to WHO, ambient air pollution causes around 4.2 million deaths annually and about 91 % of the population live in places with air pollution levels greater than the recommended safe limits [31]. While the epidemiological link between PM_2.5_ exposure and adverse health consequences is strong and circumstantial evidence that oxidative stress is involved, direct evidence is lacking. Data from the present study show convincingly that EPFR exposure induces OS in the respiratory epithelium and that many of the EPFR effects can be prevented by pre-treatment with the antioxidant, astaxanthin.

Populations exposed to ambient air pollution are exposed to EPFRs. Sampling tree leaves provides a simple way to determine the presence of EPFRs in the atmosphere. The concentrations of EPFRs collected from tree leaves in Beijing, China ranged from 2.0 × 10^17^ to 4.5 × 10^19^ spins/g [32], whereas those in Brisbane, Australia, with relatively clean air, were between 3.4 × 10^15^ to 2.6 × 10^16^ spins/g (unpublished data). EPFR concentrations measured from tree leaves in Baton Rouge, USA, mapped to prevalence of pneumonia hospitalization of children [33], demonstrating a link between ambient EPFRs and adverse health effects. The EPFR concentration (1 mg/cm^2^) used in this study was equivalent to 1.1 × 10^16^ spins/g, which suggested that the concentration we used was relevant to moderate air pollution exposure.

The results from the present study demonstrated that EPFR exposure caused dose-dependent decreases in epithelial integrity and induced mtROS generation. The demonstration that pre-treatment with astaxanthin prevented these effects, strongly suggest that they were due to EPFR-induced OS. These results are supported by Thevenot et al. (2013), who also reported that EPFRs induced OS and pulmonary pathology in the human bronchial epithelial BEAS-2B cells and *in vivo* mouse model [27]. OS in the airway epithelium has adverse impacts on cellular and mitochondrial health, which can lead to inflammation and respiratory disorders.

We have previously demonstrated [18] that individuals can be classified as “sensitive” or “resistant” to epithelial OS, based on the concentration of H_2_O_2_ required to increase epithelial permeability. In the present study, subjects in the sensitive group upregulated mechanisms designed to protect the epithelium from OS following EPFR exposure. This included increasing mitochondrial mass, activation of the aryl hydrocarbon receptor (AHR), and upregulation of pathways increasing expression of antioxidant enzymes. The inhibition of the upregulation of these defence mechanisms by astaxanthin pre-treatment, indicates that EPFR-induced OS was the likely mechanism. The one exception was the failure of the sensitive group to upregulate *MUC5AC* mRNA expression. However, even here, EPFR-induced OS is the likely mechanism, based on the prevention of this EPFR effect by astaxanthin pre-treatment. The individual pathways are discussed briefly below.

EPFR exposure increased mtDNAcn in the sensitive group. This suggested that sensitive subjects activated protective mechanism by increasing the number of mitochondria to cope with the stress. This is consistent with a study in male human that PM exposure increased mtDNAcn [34], and mtDNAcn was higher in bronchial epithelium from electronic cigarette users and smokers than never-smokers [35].

Environmental chemical exposure activates the AHR that regulates the expression of detoxifying genes including the xenobiotic-metabolizing enzyme, CYP1A1 [36]. In the present study, *CYP1A1* mRNA expression significantly increased following EPFR exposure in both sensitive and resistant groups, but more markedly so in sensitive subjects. The increase was inhibited by astaxanthin pretreatment. This is consistent with the findings in A549 human lung epithelial cells that EPFR-2-monochlorophenol (MCP) 230, exposure increased *Cyp1a1* expression and the increase was partially inhibited by an antioxidant, N-*tert*-Butyl-α-phenylnitrone [37], and a study in BEAS-2B human bronchial epithelial cells that EPFR-MCP230 exposure upregulated *Cyp1a1* mRNA expression and co-treatment with an antioxidant, α-T, inhibited the upregulation [38].

The mucin producing gene, *MUC5AC* mRNA expression declined in the sensitive group and increased slightly in the resistant group following EPFR exposure. There were no systematic differences in baseline of *MUC5AC* expression between the groups. The mucus layer is the first barrier to protect the cells from the environment. Therefore, a failure to produce mucus could result in increased access of environmental stressors to epithelial cells. The cells would be expected to respond by activating OS defence pathways. From our results, sensitive subjects failed to up-regulate *MUC5AC* expression in response to EPFR exposure, and OS defence and apoptosis pathways were activated. On the other hand, resistant subjects were able to maintain the mucus production and EPFR exposure did not cause many adverse effects on cell integrity or on gene expression. These data indicate the importance of MUC5AC in conferring protection to the epithelium.

PM exposure has been shown to induce mucus hypersecretion in some studies [39,40]. However, Kania et al. demonstrated that coal dust PM_10_ exposure in male Wistar rats decreased *MUC5AC* expression [41], a finding similar to our results. A study using BEAS-2B cells found that shorter urban PM_2.5_ exposure increased *MUC5AC* mRNA expression while expression decreased with longer exposure times and higher concentrations [42]. Thus, the differences between the studies can be the time and dose of PM exposure. In the present study, a 4-h EPFR exposure was performed, and the mRNA expression was measured at 24-h post-exposure, a dose-dependent decrease in *MUC5AC* mRNA expression was observed, this was similar to the dose effect on *MUC5AC* mRNA expression seen in the PM_2.5_ exposure on BEAS-2B cells study.

In the sensitive group, EPFR exposure activated the *SIRT1*-*FOXO3* pathway, which regulates the expression of antioxidant enzymes catalase and MnSOD (SOD2) [43]. The *SIRT1*-*p21* apoptosis pathway [44] and the *SIRT1*-*PINK1* mitophagy pathway [45] were also activated by EPFRs. In the resistant group, EPFR exposure only slightly up-regulated *PINK1* mRNA expression, which is a gene that mediates mitophagy to remove damaged mitochondria [46].

Astaxanthin pre-treatment protected against many of the adverse effects induced by EPFRs. Astaxanthin prevented EPFR-induced *MUC5AC* mRNA down-regulation, consequently, inhibited mtROS generation, apoptosis (*p21*), and mitophagy (*PINK1*), but maintained the antioxidant responses (*SIRT1*-*FOXO3*). These results are supported by a study that investigated the attenuation of astaxanthin on environmental tobacco smoke induced OS in the mice brain [47] and *in vivo* emphysema model induced by cigarette smoke [48].

Comparing the cellular responses to two oxidant stressors, EPFRs and H_2_O_2_ showed interesting results. *AOX1*, *SRXN1*, *HMOX1* and *SPINK1* were commonly up-regulated by both stressors in both sensitive and resistant groups. *AOX1*, *SRXN1* and *HMOX1* (*HO-1*) are antioxidant genes that are regulated by NRF2 [49,50]. *SPINK1* is involved in redox homeostasis by activating Nrf2 in non-small cell lung cancer [51]. These results indicated that NRF2 is a key regulator following both EPFR and H_2_O_2_ exposure.

SOD3 is located extracellularly and is a Cu–Zn-SOD type antioxidant enzyme [43]. *SOD3* was up-regulated following EPFR and H_2_O_2_ exposure in the resistant group and following H_2_O_2_ exposure in sensitive the group. This suggests that the airway epithelium responded to the exposures by promoting the antioxidant enzymes to cope with the increased OS.

*APOE*, *GPX3*, *SOD2* and *SQSTM1* were up-regulated following EPFR exposure in both groups. APOE is involved in antioxidant defence mechanisms and plays an important role in Alzheimer's disease, an OS-associated disease [52]. GPX3 and SOD2 (MnSOD) are well-known antioxidant enzymes. SOD2 is located in the mitochondria and catalyse O_2_^●−^ into H_2_O_2_. GPX3 is located extracellularly and is involved in H_2_O_2_ detoxification [53]. A study also showed exposure to the EPFR DCB230 significantly up-regulated SOD2 in BEAS-2B epithelial cells [27] supports the results of the present study. SQSTM1 is commonly aggregated with p62, they are prototype autophagy receptors and mediated by Nrf2 [54]. SQSTM1/p62 are involved in PINK1/PARKIN mediated mitophagy [55]. A study has shown that SQSTM1/p62 upregulation increased autophagy and protected retinal pigmented epithelial cell from OS [56]. In the present study, EPFR exposure up-regulated *PINK1* and *SQSTM1* expression, this suggests that the airway epithelium promoted mitophagy to remove cells affected by EPFR-induced OS.

*SEPP1* was significantly decreased following EPFR and H_2_O_2_ exposure in the sensitive group, while increasing with EPFR exposure and reducing with H_2_O_2_ exposure in the resistant group. SEPP1 has antioxidant activity, a decrease in SEPP1 can induce ROS production in human skin keratinocytes and hepatocellular carcinoma cells [57,58]. Lower SEPP1 expression was found in lung tissues in smokers and lower SEPP1 blood plasma level is linked to respiratory tract cancer development [59]. In the present study, sensitive subjects had much less *SEPP1* expression following the stress, which might be one of the reasons that mtROS levels increased. In the resistant group, EPFR exposure increased *SEPP1* expression, suggesting that resistant subjects were able to promote SEPP1-related antioxidant activity.

In the sensitive group, expression of the inflammatory cytokine *CCL5* (RANTES) increased 8-fold following EPFR exposure. CCL5 levels increase after cigarette smoke extract exposure in A549 adenocarcinomic human alveolar basal epithelial cell line [60] and higher level of CCL5 in bronchoalveolar lavage fluid was detected in ovalbumin induced asthmatic mice [61]. Data from the present study show that EPFR exposure resulted in an increase in *CCL5* expression in sensitive subjects, which may lead to inflammation, while there was no significant change in the resistant group.

Following IPA and gene-gene interaction analysis, we identified that ERK, NF-κB, NQO1, and GPX2 are closely associated with MUC5AC expression which was failed to be upregulated in sensitive subjects following EPFR exposure. Despite the EPFR-induced phosphorylation of ERK and NF-κB, it did not link to upregulation of MUC5AC in the sensitive group which is contrary with studies conducted on PM-exposed human bronchial epithelial cell line [62] and diesel exhaust particles-exposed NCI–H292 human airway epithelial cell line [39]. Phosphorylation of NF-κB was slightly reduced with astaxanthin treatment and EPFR-induced *CCL5* and *p21* were only observed in the sensitive group, these results may suggest that activation of NF-κB was associated with increased inflammation and apoptosis, similar to the study investigated the effect of cigarette smoke extract (CSE) on human bronchial epithelial cell line Beas-2B and NHBE that CSE-induced NF-κB phosphorylation, and led to inflammation and apoptosis, and the increased phosphorylation was inhibited by an anti-inflammatory compound [63].

Data from the present study demonstrate that NRF2 plays an important role in response to EPFR exposure, and EPFRs can induce multiple genes related to antioxidant response, metal ion homeostasis, and fatty acid metabolism. When ligands bind to AHR, they directly or indirectly influence NRF2 activity [64]. NRF2 regulates phase II enzymes such as GPX2 and NQO1 in xenobiotic detoxification [65]. In the present study, we observed an increase in NRF2 nuclear translocation, this suggest that NRF2-mediated antioxidant response was induced following EPFR exposure. EPFRs negatively affected the ferroptosis signaling pathway, a type of programmed cell death, which indicates that the well-differentiated respiratory epithelium from healthy individual humans can promote OS defense signaling pathway to limit the exposure-induced damages when exposure to sublethal concentration of EPFRs.

The present study has some limitations. The logistics of using well-differentiated primary respiratory epithelial cells cultured at ALI dictated a relatively small sample size, and the complexity of the studies limited the numbers of samples can be examined. Despite the limitations, the present study demonstrated significant findings.

## 5 Conclusions

This is the first study to investigate the effects of EPFR exposure in a physiological relevant model, well-differentiated primary human airway epithelium. EPFR exposure reduced epithelial barrier integrity and induced OS in the respiratory epithelium. These effects can be prevented by pre-treatment with the antioxidant, astaxanthin. Increased susceptibility to EPFR exposure is conferred by failure to upregulate the mucin gene, *MUC5AC*, expression. Different pathways were activated to response EPFR exposure in different groups. EPFR exposure in air pollution may contribute to respiratory illness such as COPD and asthma. Our results showed that astaxanthin may protect vulnerable population from air pollution exposure especially during bushfire events or people who lives in areas with higher air pollution level.

## CRediT authorship contribution statement

**Ayaho Yamamoto:** Conceptualization, Data curation, Formal analysis, Investigation, Methodology, Project administration, Validation, Visualization, Writing – original draft, Writing – review & editing. **Peter D. Sly:** Conceptualization, Formal analysis, Funding acquisition, Resources, Supervision, Writing – review & editing. **Lavrent Khachatryan:** Resources. **Nelufa Begum:** Formal analysis. **Abrey J. Yeo:** Supervision. **Paul D. Robinson:** Supervision, Writing – review & editing. **Stephania A. Cormier:** Conceptualization, Funding acquisition, Methodology, Supervision, Writing – review & editing. **Emmanuelle Fantino:** Conceptualization, Supervision, Validation, Writing – review & editing.

## Funding

This work was funded by a grant from the 10.13039/100000066National Institute of Environmental Health Sciences [P42 ES013648]; and PDS is funded by the 10.13039/501100000925National Health and Medical Research Council, Australia [1193840].

## Declaration of competing interest

None.
