# Supplementary material for: Astaxanthin protects against environmentally persistent free radical-induced oxidative stress in well-differentiated respiratory epithelium
Source: Redox Biol. 2025 Feb 9;81:103542. doi: 10.1016/j.redox.2025.103542 (PMC11875192; doi:10.1016/j.redox.2025.103542)
Supplement: Multimedia component 1 [file mmc1.docx]

**Supplementary material**

**Astaxanthin protects against environmentally persistent free radical-induced oxidative stress in well-differentiated respiratory epithelium**

Ayaho Yamamoto ^a, *^, Peter D. Sly ^a^, Lavrent Khachatryan ^b^, Nelufa Begum ^a^, Abrey J. Yeo ^a,c^, Paul D. Robinson ^a^, Stephania A. Cormier ^d^, and Emmanuelle Fantino ^a^.

^a^ Child Health Research Centre, The University of Queensland, South Brisbane, Queensland 4101, Australia

^b^ Department of Chemistry, Louisiana State University, Baton Rouge, Louisiana 70803, United States

^c^ Centre for Clinical Research, The University of Queensland, Herston, Queensland 4006, Australia

^d^ Department of Biological Sciences, and Pennington Biomedical Research Center, Louisiana State University, Baton Rouge, Louisiana 70803, United States

**
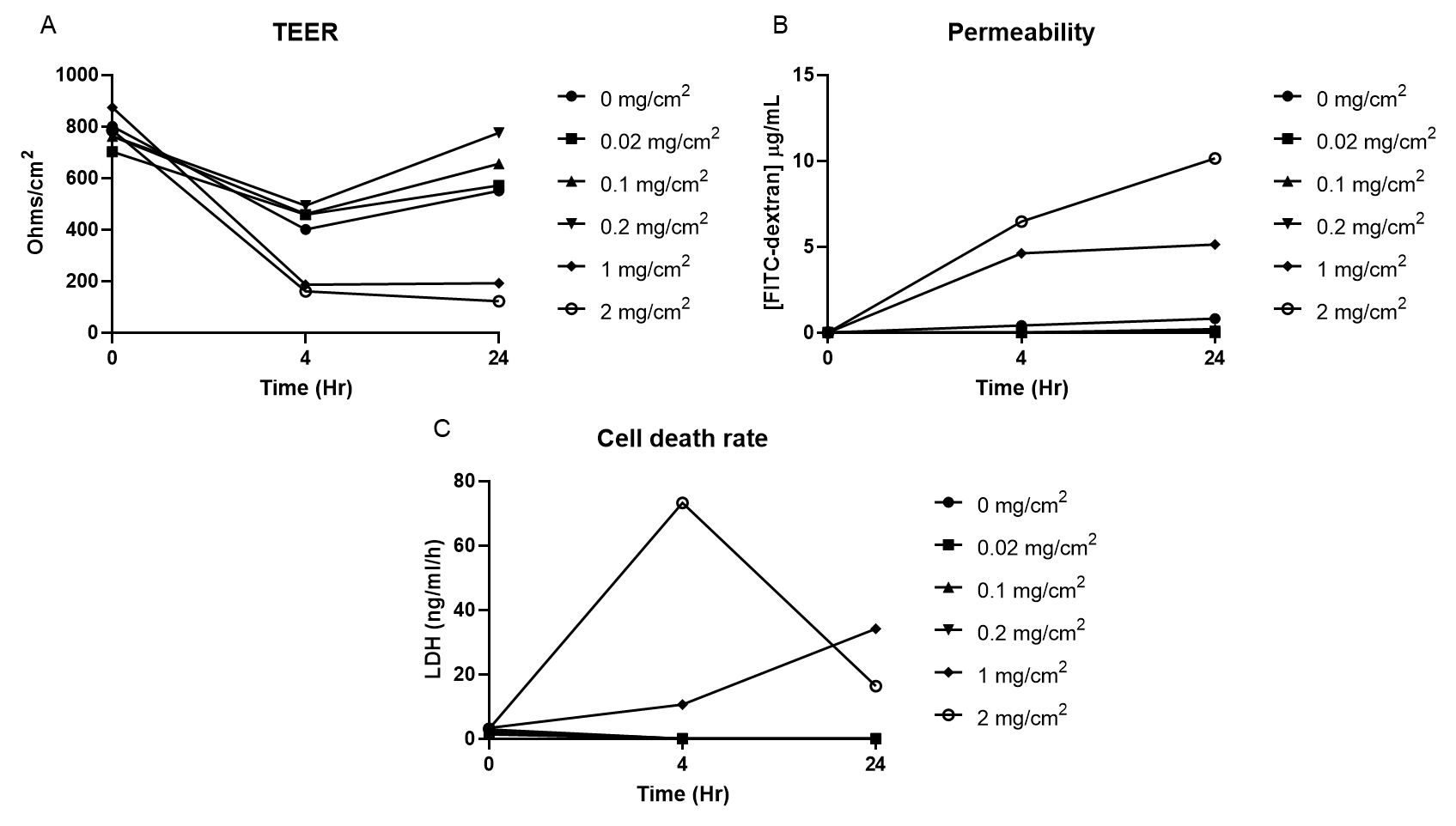
**

**Fig. S1. The effects of different concentrations of EPFRs on cell integrity.**

0, 0.02, 0.1, and 0.2 mg/cm^2^ EPFR exposure had no effect on TEER (A), permeability (B) or cell death (C). 1 and 2 mg/cm^2^ EPFR exposure caused a decrease in TEER (A) and an increase in permeability (B) and cell death (C).


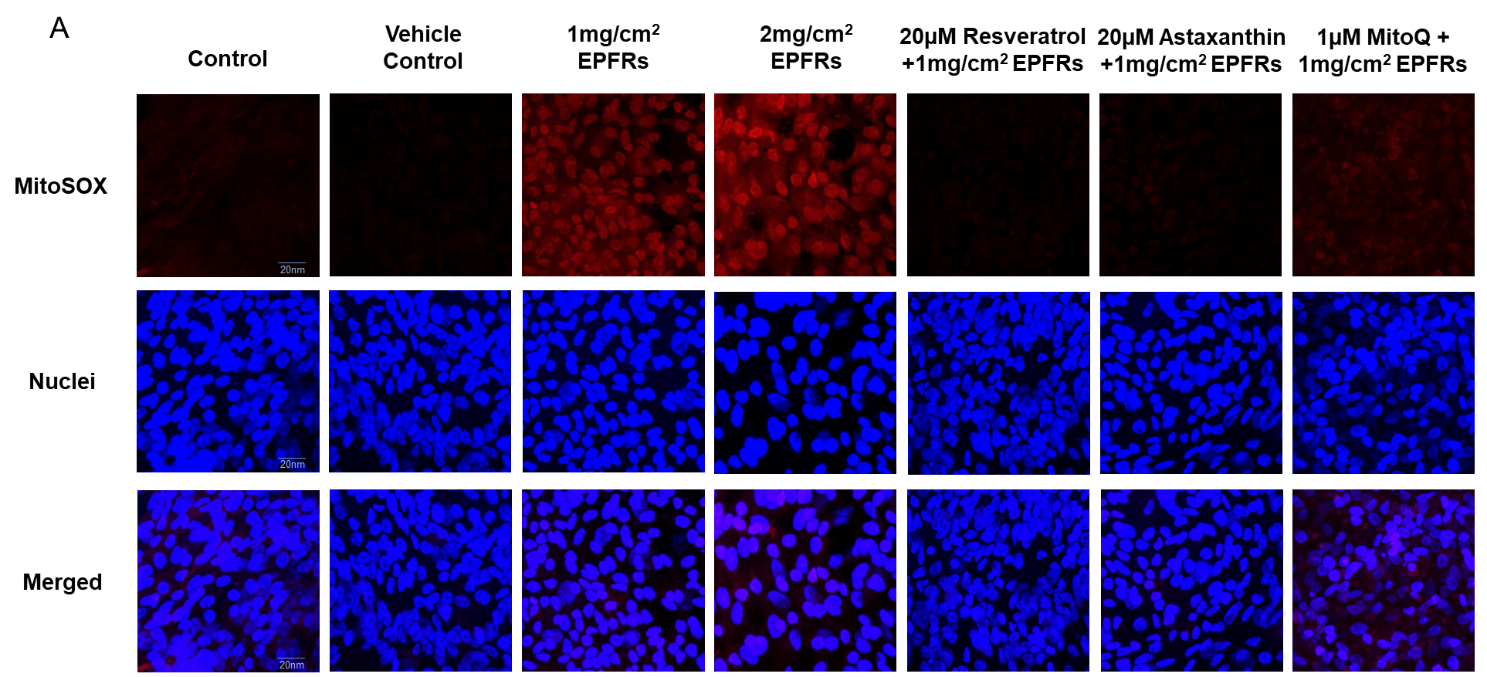


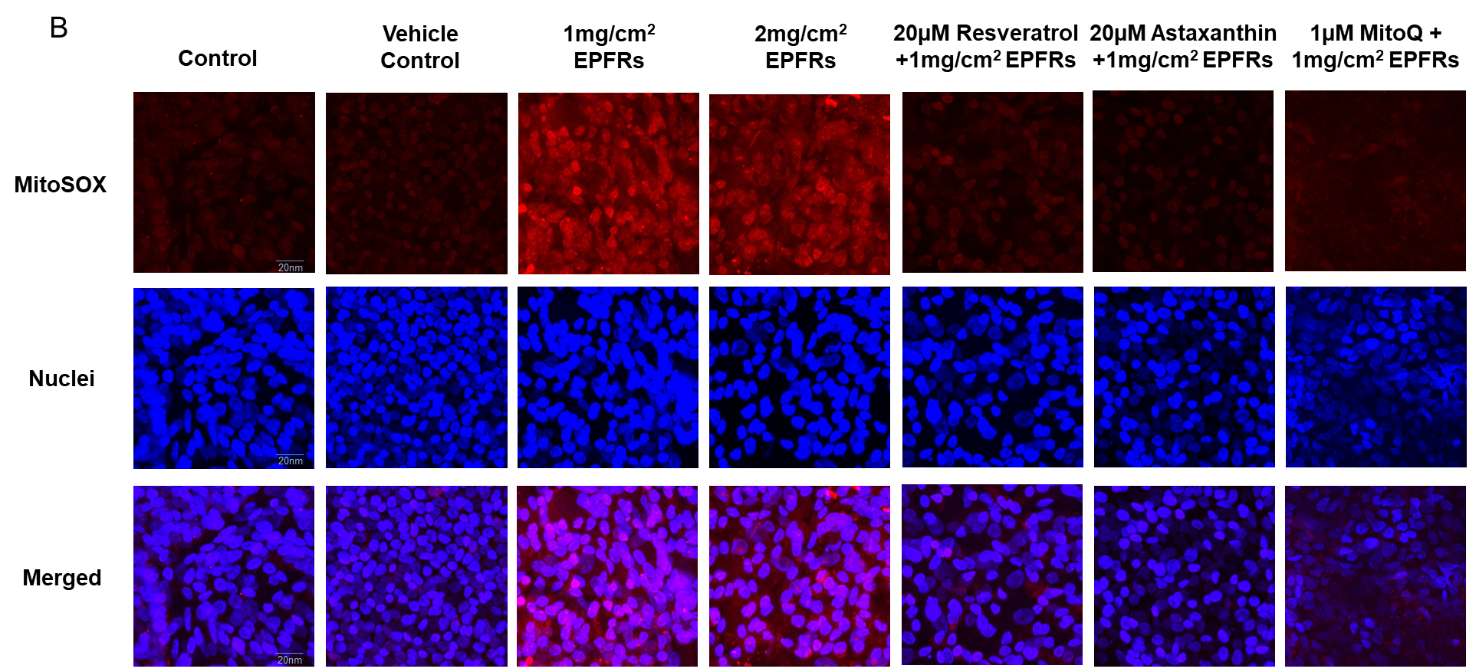


**Fig. S2. EPFR exposure induced mtROS that was inhibited by resveratrol, astaxanthin and MitoQ.**

After 4 hours EPFR exposure, MitoSOX staining were performed, confocal images were captured. Images are shown from a representative sensitive subject (A) and a resistant subject (B). Scale bar: 20 nm.


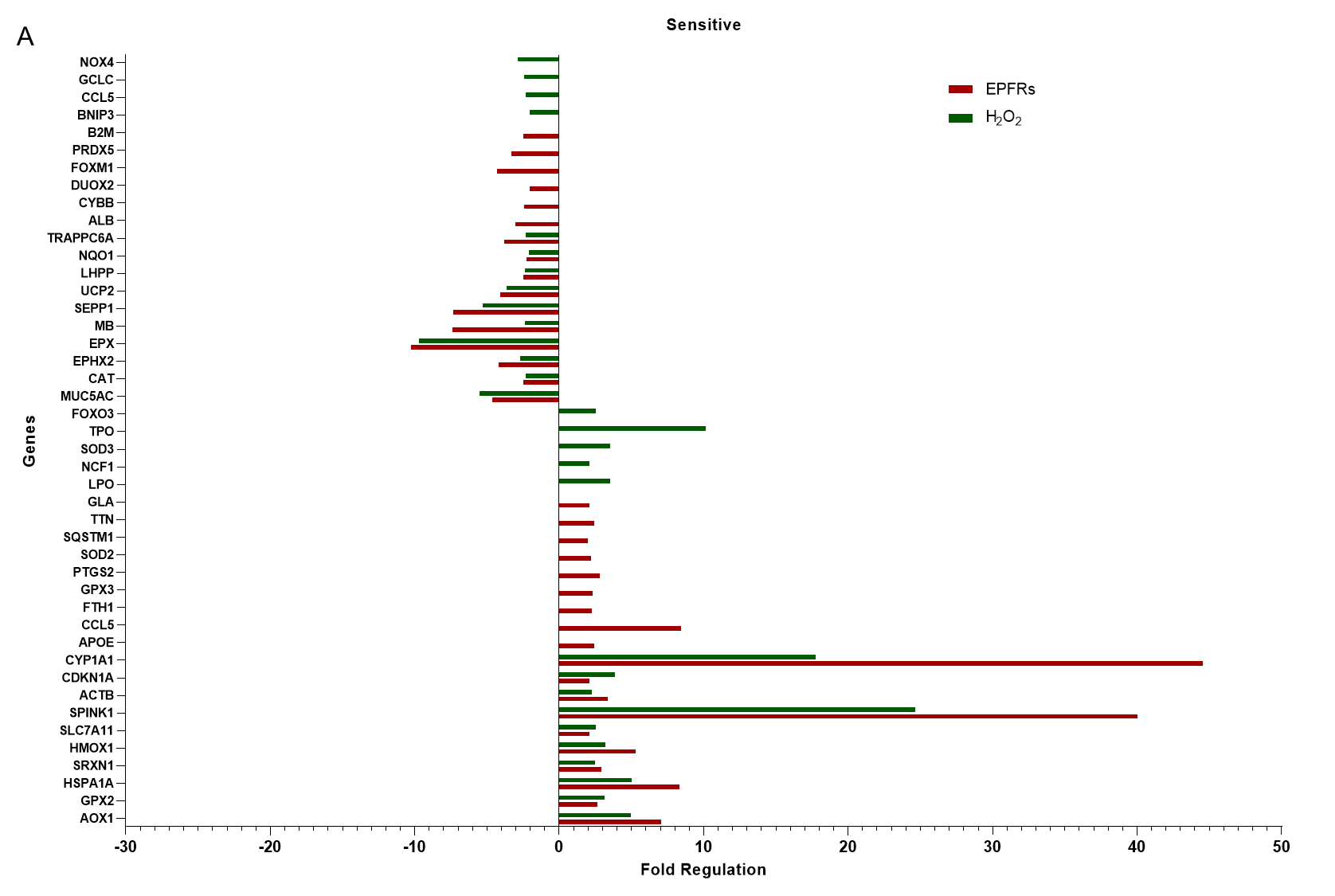


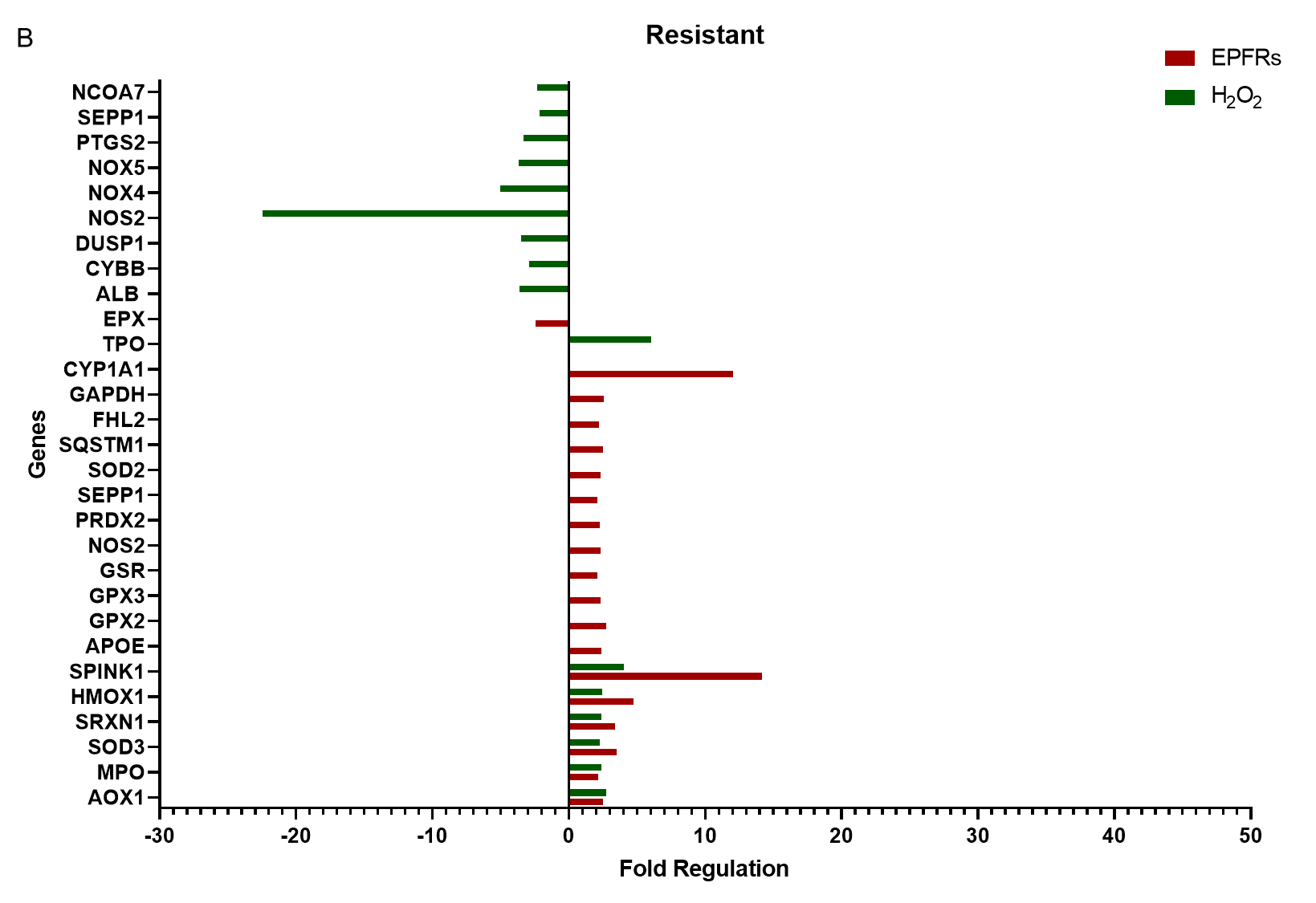


**Fig. S3. The list of genes impacted by EPFR or H_2_O_2_ exposure.**

RT² Profiler PCR Array Oxidative Stress Gene Expression analysis following 1mg/cm^2^ EPFR and 50mM H_2_O_2_ exposure in the sensitive (A), and resistant (B) groups.


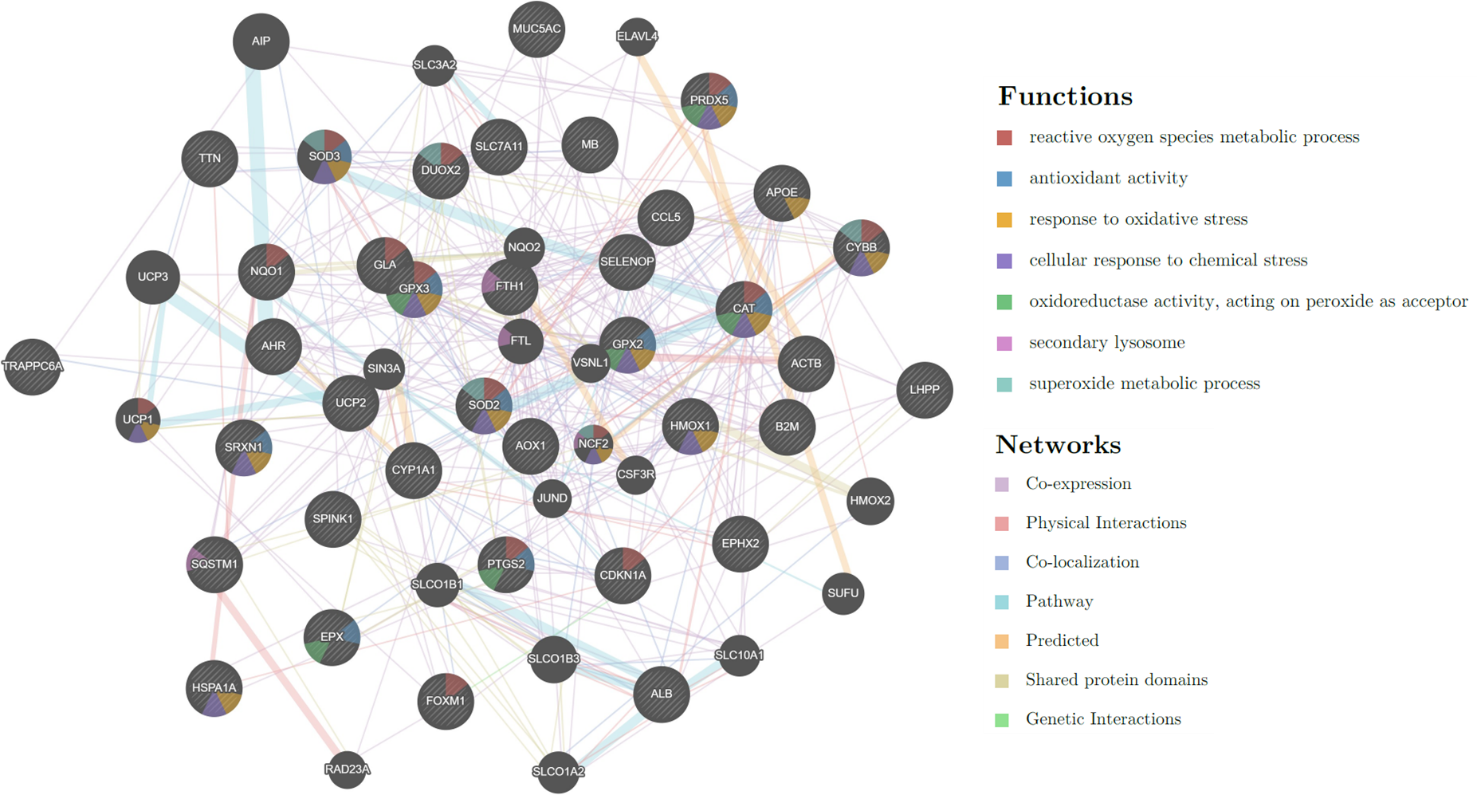


**Fig. S4. Gene interaction networks and the top functions of the genes involved in response to EPFR exposure.**

Data from PCR array were analysed using GeneMANIA database. NFE2L2: NRF2; CDKN1A: p21.


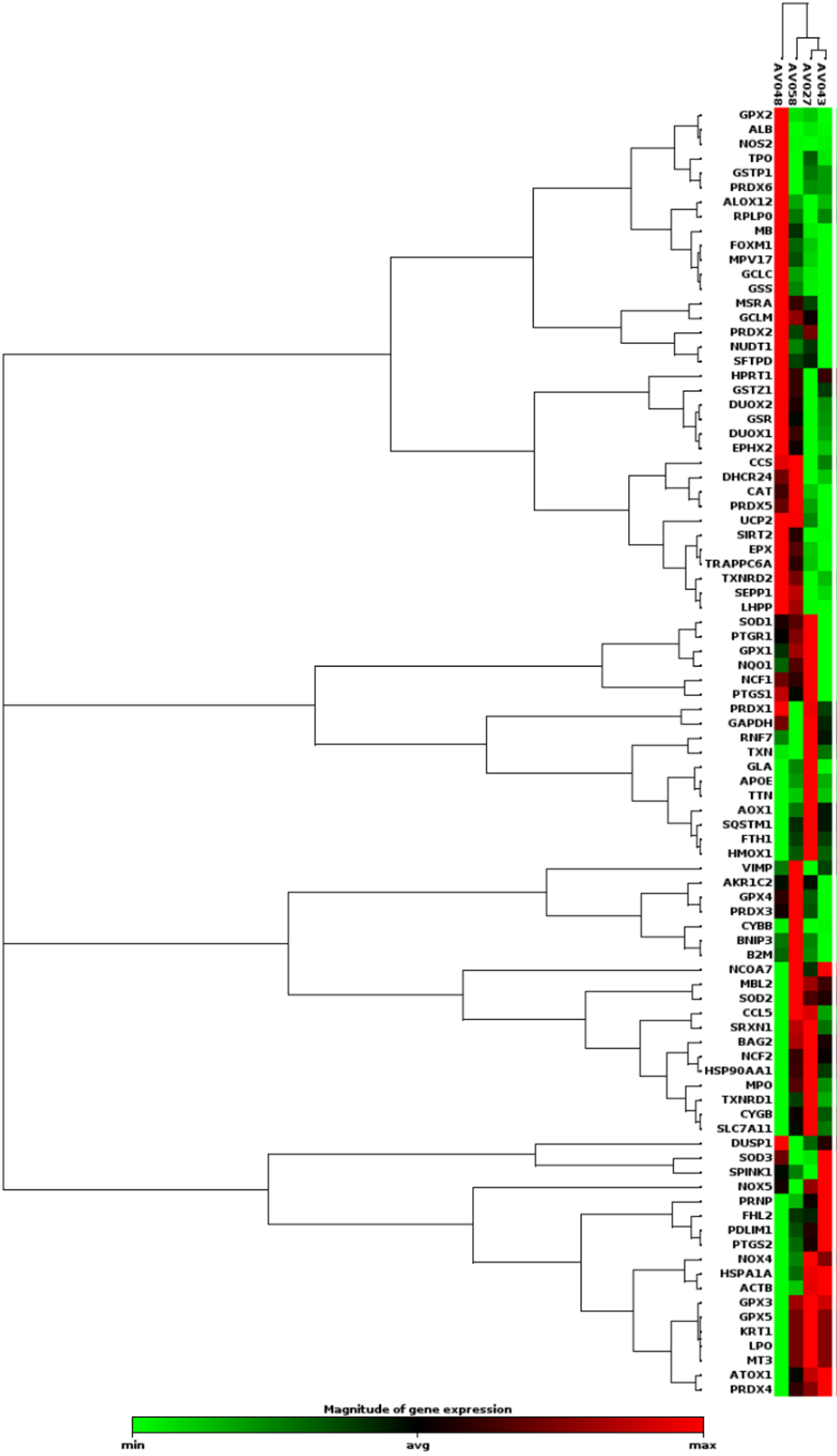


**Fig. S5. Clustergram.**

The expression of 84 oxidative stress response genes were clustered following 1mg/cm^2^ EPFR exposure. The heat map indicates the gene expression level. Sensitive group: AV027, AV043; Resistant group: AV048, AV058.

**
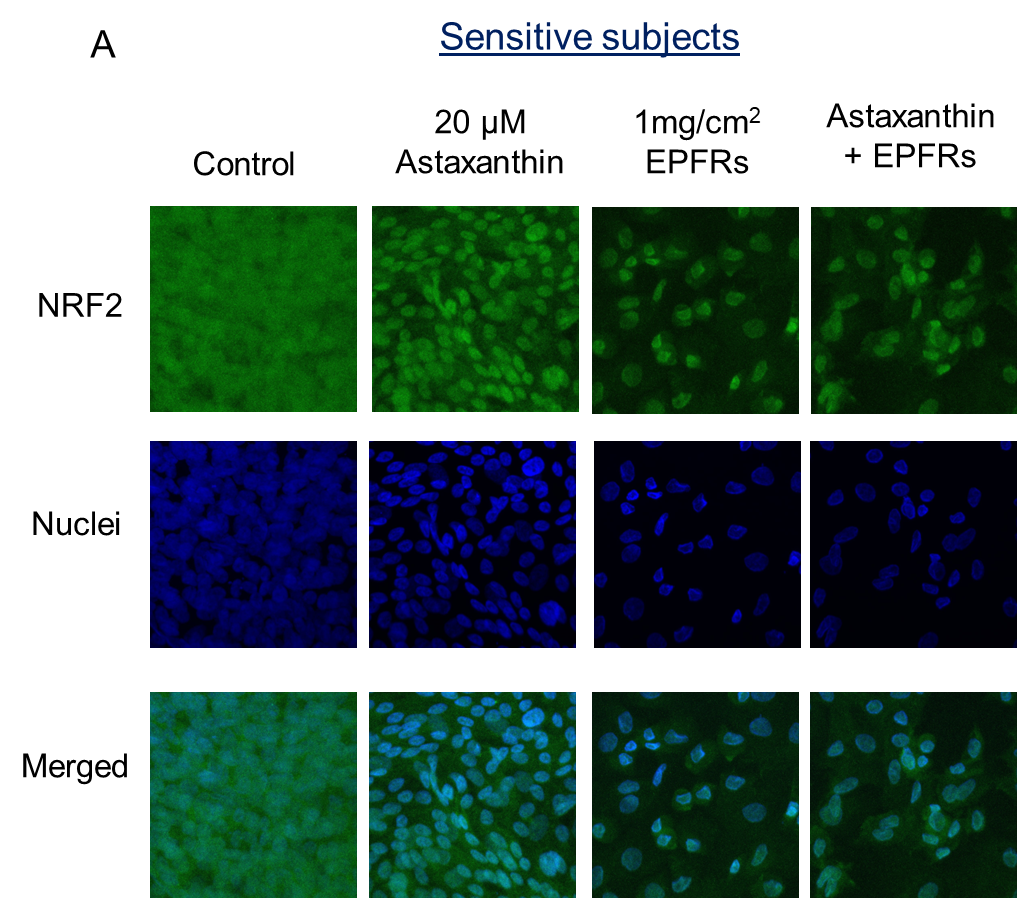

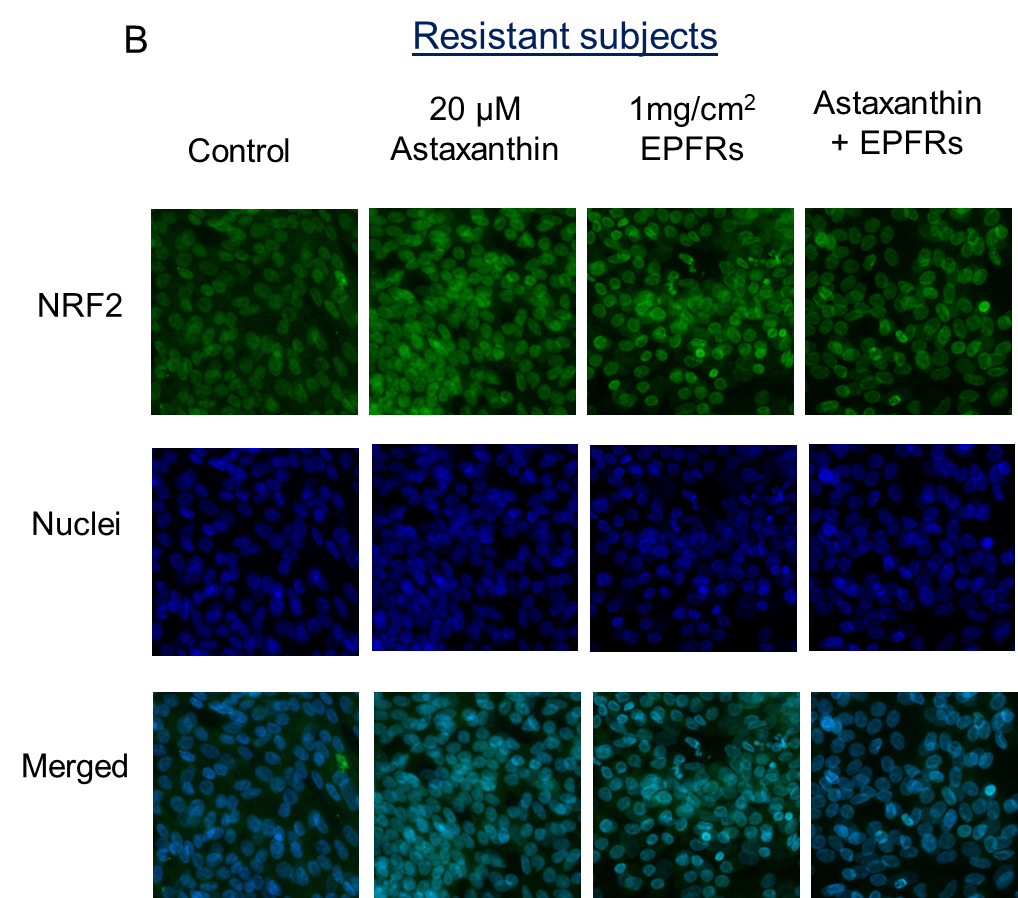
**

**Fig. S6 EPFR exposure increased NRF2 nuclear translocation.**

After 4 hours post EPFR exposure, NRF2 staining were performed, confocal images were captured. Images are shown from a representative sensitive subject (A) and a resistant subject (B).
